# Synthesis of Copper Molybdate and Its Electrochemical Sensing of Paracetamol

**DOI:** 10.7759/cureus.63925

**Published:** 2024-07-05

**Authors:** S G Krisha, S Menaka, Sherin Celshia, Muthamizh Selvamani, Vasugi Suresh

**Affiliations:** 1 Physiology, Saveetha Dental College and Hospitals, Saveetha Institute of Medical and Technical Science, Saveetha University, Chennai, IND; 2 Physiology, Saveetha Dental College and Hospitals, Saveetha Institute of Medical and Technical Sciences, Saveetha University, Chennai, IND

**Keywords:** edx analysis, dpv analysis, xrd analysis, nanoparticle, paracetamol, electrochemical sensing, copper molybdate

## Abstract

Background

In recent years, significant advancements have been made in various scientific sectors, particularly in healthcare and pharmaceutical research. This progress has been driven by the development of enhanced sensing materials and methodologies. Electrochemical sensing has become an important tool in detecting and analyzing drug molecules due to its high sensitivity, specificity, and rapid response times. Among various drugs, paracetamol, also known as acetaminophen, is widely used for its analgesic and antipyretic properties. Accurate detection of paracetamol is crucial due to its widespread use and potential for overdose, which can lead to severe liver damage. Copper molybdate (CuMoO_4_) is a transition metal oxide that has garnered attention for its excellent electrical conductivity and electrochemical stability. These properties make it a promising candidate for use in electrochemical sensors. The ability of CuMoO_4_ to act as a sensor material is enhanced by its unique structural and morphological characteristics, which can be tailored during synthesis.

Aim

This study aimed to synthesize CuMoO_4_ and investigate its electrochemical sensing capability for the detection of drug molecules, specifically paracetamol.

Materials and method

CuMoO_4_ was synthesized using a precipitation method that did not involve any surfactants. This approach was chosen to simplify the synthesis process and avoid potential contamination from surfactants. The morphology of the synthesized CuMoO_4_ nanoparticles was investigated using a field emission scanning electron microscope (FE-SEM). Energy-dispersive X-ray spectroscopy (EDX) confirmed the purity of the CuMoO_4_ nanomaterial. Structural analysis was performed using X-ray diffraction (XRD). To evaluate the electrochemical sensing capability of CuMoO_4_ for paracetamol, Differential pulse voltammetry (DPV) was employed. DPV is a sensitive electrochemical technique that can detect changes in current response corresponding to the presence of analytes.

Results

The synthesized CuMoO_4_ exhibited a rock-like structure, as revealed by FE-SEM imaging. This morphology is advantageous for electrochemical applications due to the increased surface area available for interaction with analytes. EDX confirmed the purity of the CuMoO_4_ nanomaterial, showing no significant impurities. XRD analysis indicated that the CuMoO_4_ nanoparticles were crystalline in nature, which is beneficial for consistent and reproducible electrochemical behavior. The DPV analysis demonstrated that the CuMoO_4_ sensor exhibited a linear increase in current response with increasing concentrations of paracetamol. This linear relationship indicates that CuMoO₄ is capable of detecting paracetamol effectively, with a strong and quantifiable signal response.

Conclusion

The CuMoO_4_ nanomaterial was successfully synthesized using a simple precipitation method and was characterized by its rock-like morphology and crystalline structure. Electrochemical testing using DPV showed that CuMoO_4_ has excellent sensing capabilities for detecting paracetamol, with a clear and linear current response. These findings suggest that CuMoO_4_ is a promising electrochemical sensing material for drug detection, potentially offering a reliable and efficient method for monitoring paracetamol and possibly other pharmaceuticals in various settings.

## Introduction

In recent years, the performance of electrochemical sensors has been substantially improved by the production of cutting-edge nanomaterials, enabling quick and precise analysis of target analytes. Due to their ease of use, low cost, and high sensitivity, electrochemical sensors have become potential tools for the identification and quantification of pharmacological compounds. Metal oxides have drawn a lot of interest among the many materials investigated for sensing applications because of their special physicochemical characteristics and promise for electrochemical sensing [1]. In the fields of nanotechnology and electrochemistry, copper molybdate (CuMoO_4_) has attracted a tremendous deal of interest. The search for novel materials becomes critical as the need for sensitive and effective electrochemical sensors expands across industries, and CuMoO_4_ emerges as a strong contender. In pharmaceutical research and clinical diagnostics, the electrochemical detection of drug molecules is extremely important. It makes it possible to identify and quantify the presence of drugs in biological samples, which contributes to drug discovery, therapeutic monitoring, and personalized medicine. Given that CuMoO_4_ is simple to synthesize and has properties that can be tuned, researchers are able to easily alter it to satisfy the needs of a wide range of sensing applications. Due to its intrinsic properties, CuMoO_4_ is now being explored as a material that has the potential to transform electrochemical sensing techniques [2].

With its unique crystal structure and electrochemical activity, CuMoO_4_ - a binary metal oxide made of copper (Cu) and molybdenum (Mo) - is especially well-suited for sensing applications. CuMoO_4_ is an important inorganic material that has a wide range of applications, including catalysts, photoanodes, and humidity sensors [3]. CuMoO₄ is a semiconductor widely known for its thermochromic and piezochromic properties. CuMoO_4_ possesses a unique combination of properties, including a wide bandgap, outstanding chemical stability, and a high surface area, making it an excellent candidate for various applications, including catalysis, energy storage, and sensing [4]. Its capacity for undergoing redox reactions also provides opportunities for effective electron transport, making it suitable for electrochemical sensing. The synthesized CuMoO_4_ was obtained through the ultrasound-assisted co-precipitation method, and its electrochemical determination of chloramphenicol was done. It showed excellent selectability, conductivity, and stability towards the detection of chloramphenicol [5]. The CuMoO_4_ was evaluated for the electrocatalytic activity of chloramphenicol, where the electrochemical sensor displays a wide linear range with the lowest detection limit and good sensitivity [6].

One important aspect of investigating the wide range of potential uses for CuMoO_4_ is its synthesis, especially in areas like electrochemical sensing, energy storage, and catalysis. The synthesis techniques utilized in the production of CuMoO_4_ are essential in modifying its physical and chemical properties, which in turn affect how well it functions in different applications [7]. A previous study reported a new and easy technique to synthesize CuMoO_4_ as a nanopowder without the use of any solvent and at low temperature, and it was verified for its high catalytic performance [8]. The intended characteristics and uses of the resulting CuMoO_4_ frequently influence the choice of synthesis technique, underscoring the significance of a nuanced approach in material design. Drug quality control, medical diagnostics, and pharmaceutical analysis all heavily depend on the identification and tracking of drug molecules. Conventional analytical methods for detecting drug molecules can entail laborious procedures and sophisticated equipment. Furthermore, there may be a need for extensive sample preparation with these procedures, which could result in higher expenses and possible contamination hazards. To overcome these obstacles, it is crucial to provide sensitive, focused, and quick sensing platforms for drug molecule detection [9]. Because of their ease of use, high sensitivity, and possibility for real-time monitoring, electrochemical sensing techniques have become highly effective tools in the detection of drug molecules. These methods are based on measuring the current or potential changes brought about by the electrochemical reactions that happen at the surface of the sensing material in response to the target analyte. The drug molecule used for sensing is paracetamol. Paracetamol (acetaminophen or para-hydroxyacetanilide) is a non-opioid analgesic and antipyretic agent used to treat fever, mild to moderate pain, and arthritis in adults and children. Paracetamol has adverse effects such as low blood pressure, heart attack, gastrointestinal bleeds, liver injury, and fulminant hepatic failure, and its overdose may result in severe metabolic acidosis [10]. The nanosheet-assembled lindgrenite microflowers not only showed a favorable linear range and high sensitivity but also demonstrated practical application and reliability towards human serum sample detection [11]. Paracetamol was successfully detected in the presence of 4-aminophenol through the use of a molybdenum trioxide (MoO₃) nanobelt-graphene oxide (GO) composite modified carbon paste electrode (CPE) with desirable linear rates [12]. The palladium/graphene oxide (Pd/GO) nanocomposite can be utilized as an improved sensing platform for the electrochemical determination of paracetamol, as it exhibits better electrocatalytic activity during the oxidation of paracetamol with a larger peak current and a lower oxidation potential [13]. A titanium-sol-based sensor detected paracetamol in environmental water with precision [14].

In this study, we focus on the synthesis of CuMoO_4_ and investigate its potential as an electrochemical sensing material for paracetamol drug molecules. This study contributes to providing techniques for minimizing the adverse effects of paracetamol drugs.

## Materials and methods

The materials used in the experiments included copper acetate monohydrate, sodium molybdate, distilled water, copper molybdate, ethanol, and a muffle furnace.

Synthesis of CuMoO_4_ nanoparticles

**Figure 1:**
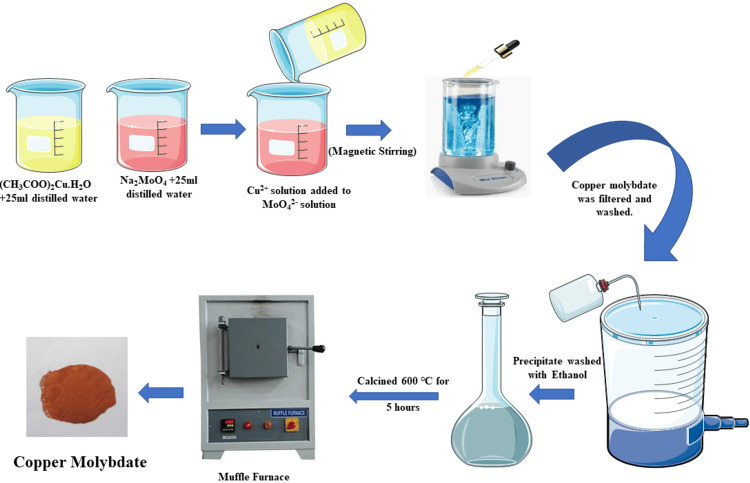
Synthesis scheme of CuMoO₄ Copper molybdate: CuMoO₄ Image Credit: Krisha SG and Muthamizh Selvamani

As depicted in Figure 1, CuMoO_4_ nanoparticles were synthesized using a precipitation method without any surfactant. The starting materials used were cupric acetate monohydrate [(CH_3_COO)_2_Cu·H_2_O], labeled as solution A, and sodium molybdate [Na_2_MoO_4_], labeled as solution B. (CH_3_COO)_2_Cu·H_2_O was dissolved in 25 ml of double-distilled water to form a Cu^2+^ solution. Na_2_MoO_4_ was dissolved in 25 ml of double-distilled water to form a MoO₄²⁻ solution.

The Cu^2+^ solution (solution A) was added dropwise to the MoO₄²⁻ solution (solution B) under continuous magnetic stirring at room temperature. This slow addition helped achieve a homogeneous mixture and prevented localized supersaturation, which could lead to uneven precipitation. After the complete addition of the Cu^2+^ solution to the MoO₄²⁻ solution, CuMoO_4_ nanoparticles precipitated out of the solution. The resulting precipitate was collected by filtration.

The collected precipitate was washed three or four times with double-distilled water to remove any unreacted starting materials and impurities. Subsequently, the precipitate was washed with ethanol to further purify it and remove any remaining water. The washed precipitate was air-dried at ambient temperatures for two days. The dried CuMoO_4_ was then subjected to calcination in a muffle furnace at 600 °C for five hours. Calcination facilitated the removal of any organic residues and the formation of a well-crystalline CuMoO_4_ phase.

Electrochemical sensing

The electrochemical sensing experiments were conducted using a three-electrode system consisting of a working electrode (glassy carbon electrode, GCE), a reference electrode (silver chloride, AgCl), and a counter electrode (platinum wire). The GCE, which serves as the primary interface for electrochemical interactions, was polished sequentially with alumina pastes of different particle sizes (1 μm, 0.3 μm, and 0.05 μm) to achieve a mirror-like finish. This step ensured a smooth and clean electrode surface, which is crucial for reproducible electrochemical measurements. After polishing, the GCE was cleaned by ultrasonication in double-distilled water for a few minutes to remove any adhering alumina particles and other contaminants.

A CuMoO_4_ suspension was prepared by dispersing 5 mg of the synthesized CuMoO_4_ nanoparticles in 10 ml of ethanol. This mixture was subjected to ultrasonic agitation for 20 minutes to ensure a uniform dispersion of the nanoparticles. The cleaned GCE was then coated with 10 μL of the CuMoO_4_ suspension using the drop-coating method. This method involves placing a small drop of the suspension onto the GCE surface and allowing it to spread evenly. The coated electrode was air-dried to allow the ethanol to evaporate, leaving a thin film of CuMoO_4_ nanoparticles on the GCE surface. 

The modified GCE, along with the reference and counter electrodes, were immersed in a glass cell containing the electrolytic solution. The specific composition of the electrolytic solution was chosen based on the intended electrochemical investigations. This setup created an ideal environment for studying the electrochemical properties of CuMoO_4_ and its potential applications, such as in sensors or catalytic processes. By meticulously preparing and modifying the electrodes and ensuring the correct setup of the electrochemical cell, reliable and reproducible electrochemical measurements could be achieved, enabling the detailed study of CuMoO_4_’s properties and behavior in various applications.

## Results

X-ray diffraction analysis of CuMoO_4_

**Figure 2:**
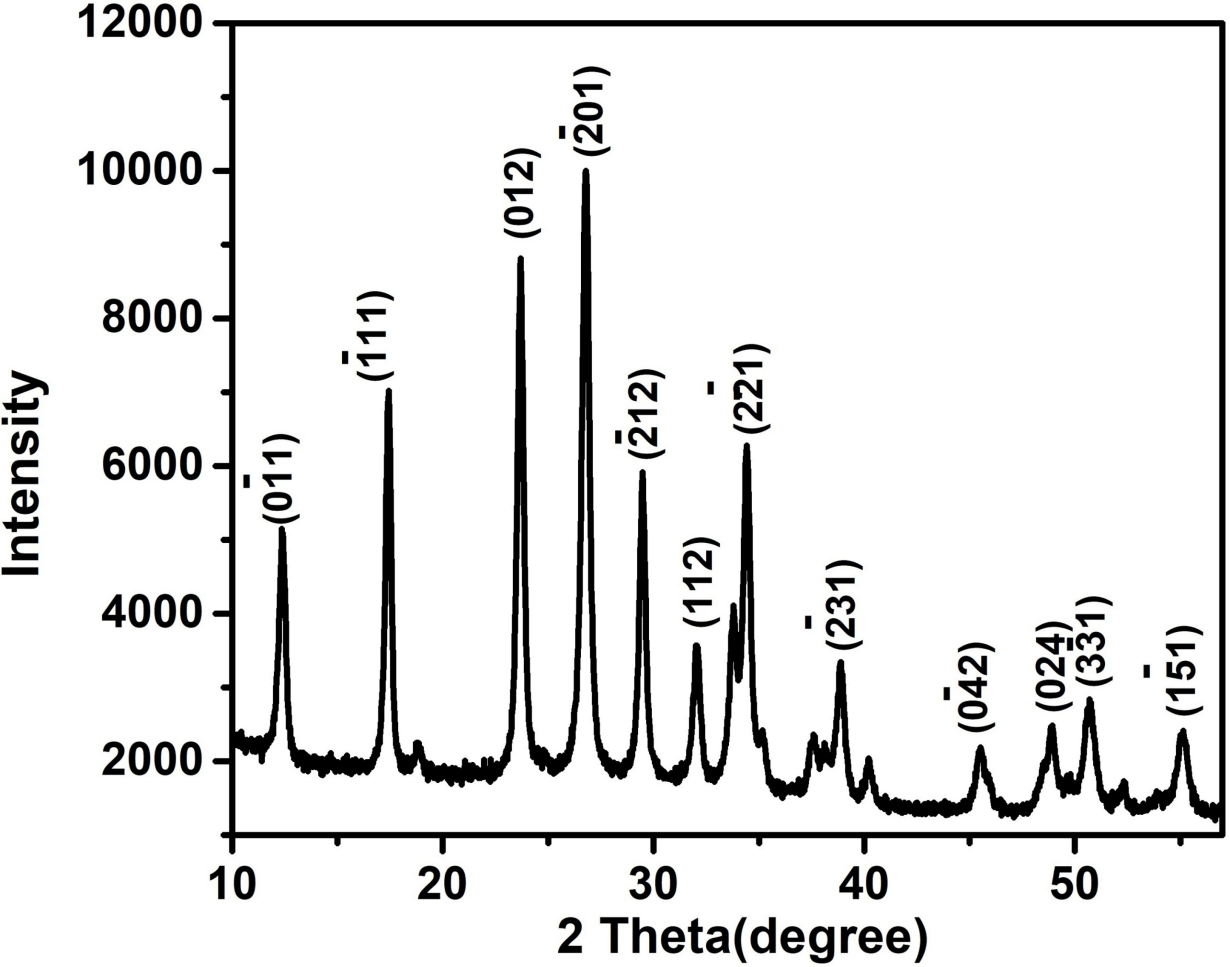
An XRD pattern of the CuMoO₄ X-ray diffraction (XRD), Copper molybdate (CuMoO₄)

The crystalline phase and nature of the synthesized CuMoO_4_ were analyzed by X-ray diffraction (XRD). The diffraction peaks of CuMoO_4_ matched well with the standard reference (JCPDS card No. 31-0449). CuMoO_4_ typically exists in two forms: the α-phase, which is orthorhombic, and the β-phase, which is triclinic. The exact phase obtained can depend on the synthesis conditions, such as temperature and method. XRD analysis can help determine the specific crystal phase of the synthesized CuMoO_4_. In Figure 2, it shows the peaks at 2θ: 12.37°, 17.36°, 23.72°, 26.70°, 29.45°, 32.11°, 34.44°, 38.90°, 45.37°, 48.88°, 50.79°, and 53.58° correspond to (0-11), (-111), (012), (-201), (-212), (112), (2-21), (-231), (0-42), (024), (3-31), and (1-51) planes of triclinic CuMoO_4_, respectively. The high intensity and sharp diffraction peaks of the synthesized CuMoO_4_ indicate its crystalline nature.

Morphological analysis of CuMoO_4_

**Figure 3:**
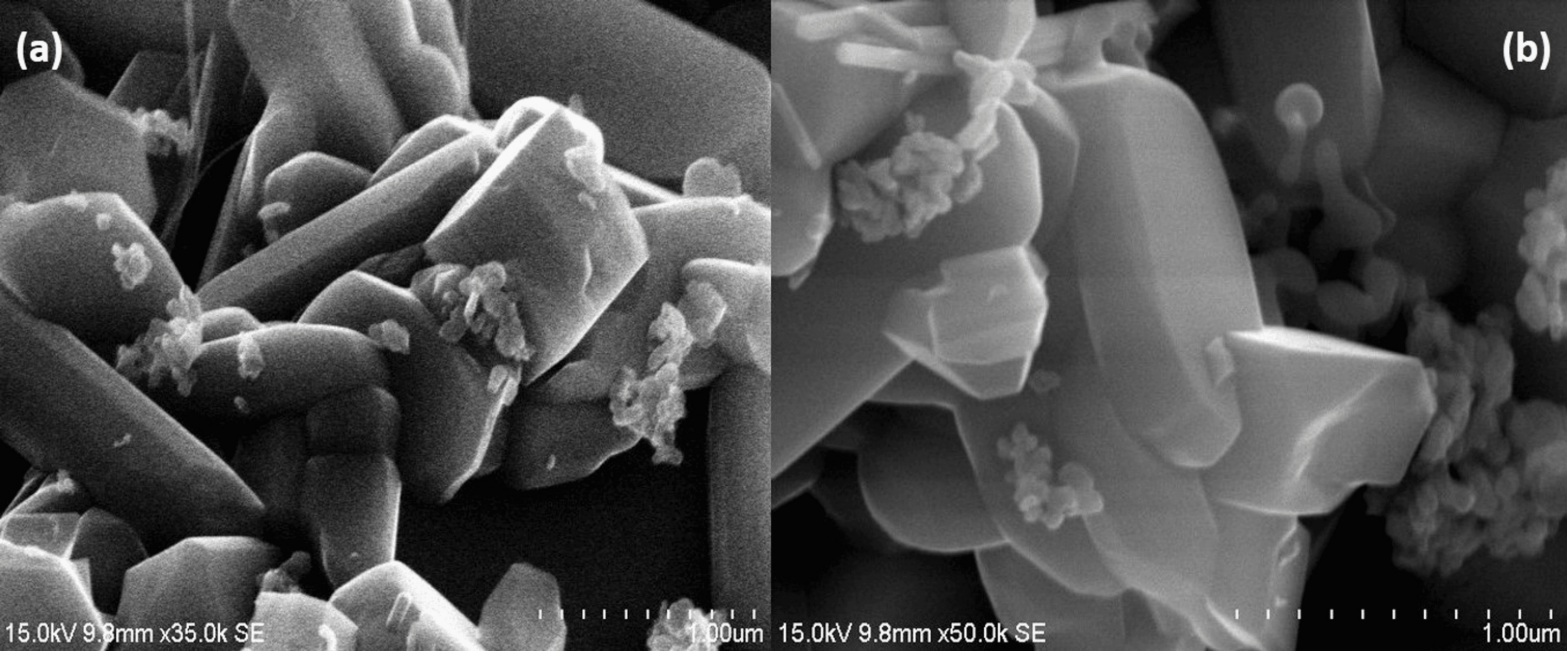
(a, b) FE-SEM images of CuMoO₄ synthesized through hydrothermal treatment Field emission scanning electron microscope (FE-SEM), copper molybdate (CuMoO_4_)

The synthesized CuMoO_4_ was then studied for its morphology through field emission scanning electron microscopy (FE-SEM). In Figure 3(a,b), FE-SEM micrographs of CuMoO_4_ showed a rock-like structure and the presence of CuMoO_4_ nanoparticles in terms of nanometers. Energy dispersive X-ray spectrometry (EDX) was used to analyze the chemical composition and purity of CuMoO_4_. As seen in Table 1, the only elements present are copper (Cu), molybdenum (Mo), and oxygen (O), which proves that the synthesized CuMoO_4_ is in pure form. Additionally, carbon (C) and nitrogen (N) were not identified in the EDX spectrum, confirming that the synthesized nanoparticles are free of impurities.

**Table 1:** EDX analysis result of the synthesized CuMoO₄ particles EDX: energy-dispersive X-ray, CuMoO_4_: copper molybdate

Element	Weight%	Atomic%
O K	25.13	62.40
Cu L	31.24	19.53
Mo L	43.63	18.07
Totals	100.00	

Differential pulse voltammetry analysis of paracetamol drug

**Figure 4:**
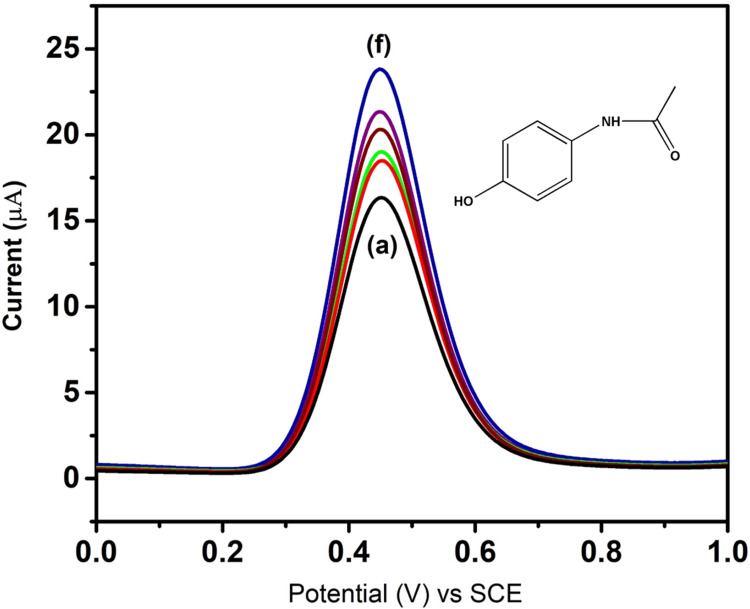
Differential pulse voltammetry response for paracetamol detection using CuMoO₄ CuMoO_4_: copper molybdate

The differential pulse voltammetry (DPV) analysis of drug molecules was conducted using a CuMoO_4_-modified GCE with varying concentrations of paracetamol (0.2 to 0.7 μM). Paracetamol exhibited distinct current responses ranging from 16 to 25 μA at an applied potential of 50 mV/s. Figure 4 shows the DPV curve illustrating changes in peak current corresponding to the redox reactions of paracetamol at the CuMoO_4_ electrode. Each peak represents a different concentration of paracetamol. The gradual linear increase in current response at different potentials indicates the CuMoO_4_ sensing capability towards the drug molecule with successive additions of paracetamol.

## Discussion

We successfully synthesized CuMoO_4_ using a simple and cost-effective method. The synthesis process plays a critical role in determining the size, morphology, and crystalline structure of the nanoparticles, which influence their electrochemical properties [15]. Earlier studies demonstrated the biosynthesis of CuMoO_4_ using the precipitation method, and its antimicrobial activities were tested against various pathogens [16].

The physical characteristics of CuMoO_4_ were studied using FE-SEM, EDX, and XRD. XRD analysis of CuMoO_4_ in this study confirmed its crystalline structure, with diffraction peaks matching standard JCPDS no. 31-0449. The peaks at 2θ:12.37°, 17.36°, 23.72°, 26.70°, 29.45°, 32.11°, 34.44°, 38.90°, 45.37°, 48.88°, 50.79°, and 53.58° correspond to (0-11), (-111), (012), (-201), (-212), (112), (2-21), (-231), (0-42), (024), (3-31), and (1-51) planes of triclinic CuMoO_4_, respectively. The prominent peaks seen in Figure 2 indicate its crystalline nature. FE-SEM images of CuMoO_4_ nanoparticles (Figure 3(a,b)) provided insights into their rock-like structure, with particles being nanometers in size as indicated by the image scale. EDX analysis (Table 1) confirmed the compound's purity, showing only copper, molybdenum, and oxygen elements present, demonstrating the synthesized nanoparticle's purity. All these physical surface characteristics underscore CuMoO_4_'s potential as an exceptional biochemical sensor. CuMoO_4_ has also shown utility in other fields. Its dominant nonlinear refraction (NLR) in CuMoO_4_/polymethyl methacrylate (PMMA) makes it useful for applications such as lasers and optical switches [17].

CuMoO_4_ has potential applications in ultralow-temperature cofired ceramic (ULTCC) due to its low relative permittivity and sintering temperature [18]. A CuMoO_4_-graphene nanocomposite shows promise for high-performance supercapacitors [19]. Its negative thermal expansion contributes to its good luminescent and thermal cycling properties, expanding its potential applications [20]. CuMoO_4_'s sensing capability for paracetamol was analyzed using DPV, yielding satisfactory results. Current changes were recorded across varying potentials. In the plot, 'a' represents the initial drug concentration detected by CuMoO_4_-modified GCE at 0.5 mV, with its corresponding current response recorded. This process was repeated for higher drug concentrations, with 'F' indicating the final drug concentration. Recorded current responses ranged from 16 to 25 μA, showing paracetamol's linear response within this range (Figure 4). Coupled with its unique surface characteristics, CuMoO_4_'s electrochemical activity forms a robust foundation for effective electrochemical sensing platforms [21]. Cysteine-capped CuMoO_4_ nanoclusters detected methotrexate [22], highlighting CuMoO_4_'s redox properties in sensing paracetamol [23]. Oleic acid-stabilized copper/cuprous oxide (Cu/Cu_2_O) nanoparticles also sensed paracetamol [24], while single-walled carbon nanotube/carbon composite electrode (SWCNT/CCE) detected acetaminophen with a linear response range of 0.2-100.0 μM in DPV [25]. Paracetamol's adverse effects include gastrointestinal bleeding, asthma, and hypertension [26].

We were able to successfully sense paracetamol using copper molybdate. CuMoO_4_ might help us to efficiently identify and biochemically sense paracetamol as its target drug molecule. Future research can investigate the sensing capabilities of CuMoO_4_ for environmental pollutants or biomarkers. The incorporation of CuMoO_4_ into composite materials can be investigated as it can provide synergistic effects, enhance sensing properties, and increase selectivity towards drug molecules. Testing the CuMoO_4_ in real-world samples such as biological fluids or pharmaceutical formulations can also be done.

Limitation

Limitations include potential challenges in controlling particle size and morphology during synthesis, which can affect the reproducibility and consistency of the CuMoO_4_ nanoparticles. Additionally, the electrochemical performance may be influenced by the presence of interfering substances in real samples. There is also a need for further optimization to enhance the selectivity and stability of the CuMoO_4_ sensor. The long-term stability and repeatability of the sensor in various environmental conditions need to be addressed. Moreover, scaling up the synthesis process while maintaining quality and performance poses another challenge.

## Conclusions

The synthesis of CuMoO_4_ nanoparticles was successfully accomplished, with their structural and morphological characteristics confirmed by XRD, FE-SEM, and EDX analyses. XRD analysis validated the crystalline structure of the nanoparticles, while EDX confirmed their purity, revealing only the presence of copper, molybdenum, and oxygen elements. FE-SEM images displayed a distinct rock-like morphology for the CuMoO_4_. These comprehensive analyses indicate that the synthesized CuMoO_4_ nanoparticles are free from impurities and possess a well-defined structure. Furthermore, the electrochemical properties of CuMoO_4_ were found to be highly favorable, suggesting their suitability for sensing applications. Specifically, CuMoO_4_ demonstrated excellent performance in detecting paracetamol drug molecules, showcasing high sensitivity and specificity. This makes CuMoO_4_ a promising candidate for the development of efficient electrochemical sensors in pharmaceutical applications. Overall, the study highlights the potential of CuMoO_4_ nanoparticles in the field of electrochemical sensing, particularly for detecting pharmaceutical compounds like paracetamol.
